# Sciatica from a Foraminal Lumbar Root Schwannoma: Case Report and Review of Literature

**DOI:** 10.1155/2012/142143

**Published:** 2012-01-15

**Authors:** Tarush Rustagi, Siddharth Badve, Aseem N. Parekh

**Affiliations:** Department of Orthopaedics, Topiwala National Medical College & B.Y.L. Nair Hospital, Mumbai 400008, India

## Abstract

Sciatica is commonly caused by lumbar prolapsed intervertebral disc (PID) and other spinal lesions. Uncommon causes like nerve root schwannoma are rarely considered in the differential diagnosis of sciatica. Spinal schwannomas occur both sporadically and in association with neurofibromatosis type 1 (NF1; von Recklinghausen's disease). This case report describes lumbar foraminal schwannoma as an unusual cause of radiculopathy, presenting clinically as a lumbar disc prolapse. The diagnosis was confirmed on MRI scan. Patient had complete symptomatic recovery following surgical enucleation of the tumour mass from the L5 nerve root. This case report is of particular interest as it highlights the diagnostic confusion, which is bound to arise, because the clinical presentation closely mimics a lumbar PID. This often leads to delay in diagnosis and “failure of conservative treatment.”

## 1. Introduction

Radiculopathy along the sciatic nerve distribution is a common spinal condition, most cases being attributed to lumbar degenerative conditions and discogenic pathologies [1]. Rare extraspinal and spinal conditions presenting clinically as a prolapsed intervertebral disc have been mentioned in the literature. Schwannomas are uncommon, comprising approximately 3% of all spinal tumours. Such schwannomas presenting with radiculopathy are often missed clinically. This is attributed to the sheer number of the cases having a classical presentation of a disc disease that we come across.

## 2. Case Report

A 40-year-old male, plumber by occupation, presented with complaints of radicular pain along the buttock, posterior aspect of thigh and calf, along the course of the right sciatic nerve of three months duration. Except for a restricted right-sided straight leg raising test, the neurological examination showed no abnormality. As the occupation of the patient involved heavy weight lifting activities, a provisional clinical diagnosis of lumbar disc syndrome was made.

Patient was started on symptomatic treatment consisting of a brief period of rest, restriction of his heavy activities, and nonsteroidal anti-inflammatory analgesics. Radiographs of the lumbosacral spine did not reveal any significant changes.

Patient reported no relief of his symptoms over a period of six weeks, with persistence of tension signs, and was out of his job during this period.

**Figure 1:**
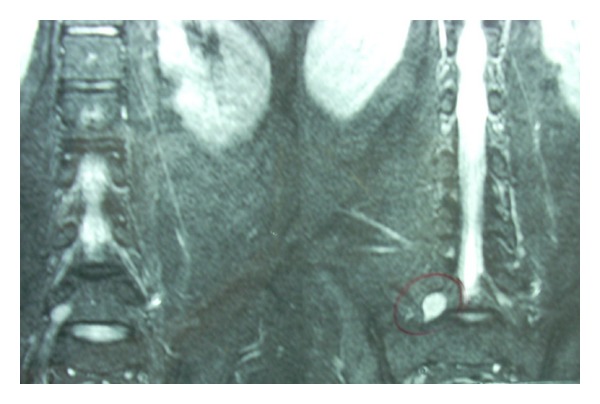
MRI T2W image (coronal) shows hyperintense nerve sheath tumour involving the right sided L5 nerve root.

**Figure 2:**
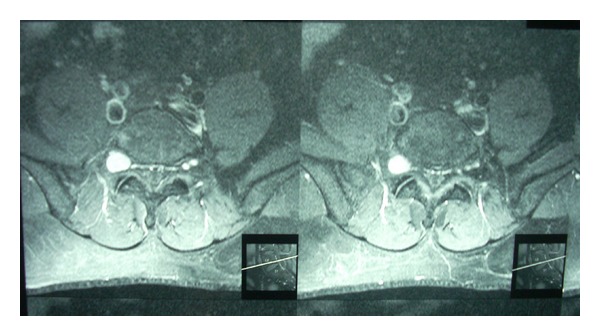
MRI T2W image (axial) shows hyperintense nerve tumour of the L5 nerve root in the L5-S1 neural foramina.

An MRI done at this stage suggested a small nerve sheath tumour of the right exiting L5 nerve root in the L5-S1 neural foramen measuring 1.7 × 1.4 × 1.3 cm in dimensions (Figures 1 and 2). The disco-vertebral structures were intact.

**Figure 3:**
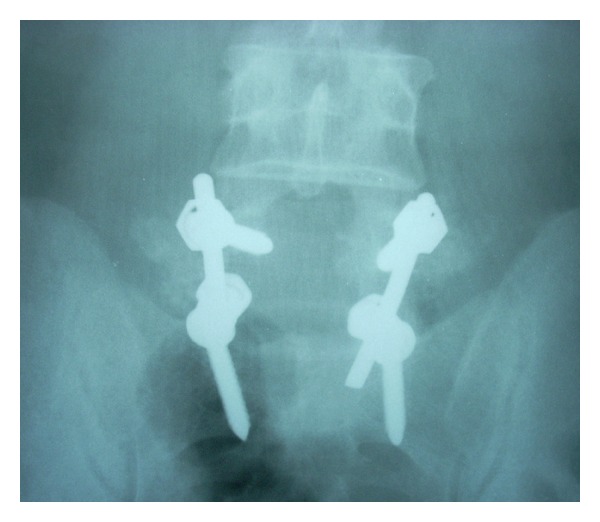
One-year postoperative L5 laminectomy, right-sided foraminotomy, and instrumentation, anteroposterior view.

**Figure 4:**
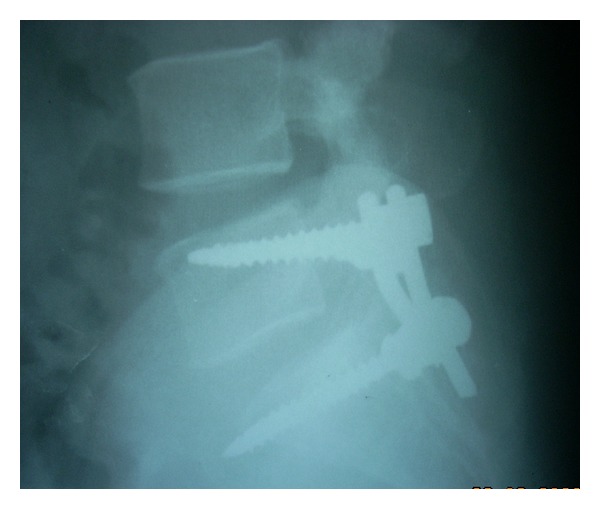
One-year postoperative, lateral view.

After appropriate counseling and workup, the patient was offered a posterior surgery. An L5 laminectomy and right-sided foraminotomy were done to adequately expose the tumour mass. A round mass involving the L5 nerve root in the foraminal region was isolated. The tumour was separated from the remaining neural tissue by careful dissection along the capsule. In view of the breach in the pars interarticularis during exposure of the foraminal region, stabilization of the spine was achieved by instrumented posterolateral fusion between L5 and S1 (Figures 3 and 4). Frozen section and histological examination of the excised tissue suggested a nerve sheath cell tumour.

Postoperatively, the patient had immediate relief of radicular symptoms and free straight leg raising test. He developed grade3 (MRC scale) weakness involving the right gluteus medius and extensor hallucis longus and sensory paraesthesia over the L5 dermatome. The possibility of this anticipated complication was explained to the patient before surgery. Patient was ambulated on the third day in a lumbar corset. The postoperative course was uneventful.

At the 3-month followup, the weakness and paraesthesia involving the right L5 root had recovered completely and the patient had resumed his occupational activities with slight restriction of heavy work.

Detailed histological examination of the excised mass indicated a neurilemoma consisting of encapsulated tumour, composed of fascicles of spindle cells arranged in a whorled pattern with nuclear palisading.

## 3. Discussion

This case highlights one of the rare intraspinal causes of sciatica due to a foraminal lumbar root schwannoma, mimicking a lumbar disc prolapse syndrome. Schwannomas are tumours of the myelin-producing Schwann cells of the nervous system. They are also known as neurilemmomas, a synonymous. Sometimes the term neurofibroma is used to describe a schwannoma, but the two are different. Neurofibromas, as seen in neurofibromatosis, invade the nerve root, becoming inseparable from it, thereby making complete surgical excision impossible without damage to the nerve itself [2]. Schwannomas, by contrast, do not invade the underlying nerve root and can be usually excised without creating neurological deficit [2]. Diagnosis of this condition is often not reached clinically. An MRI scan with contrast and finally a histopathological section are required for the diagnosis.

Incidence of foraminal tumours is 1–5%; schwannomas being the most common (60%) [2]. There have been reports of neurilemoma involving the sciatic nerve in the extraforaminal course in the pelvis, thigh and peripheral portion presenting as sciatica [3, 4]. There is paucity of literature describing foraminal neurilemoma involving the lumbar root presenting as sciatica [2, 5, 6].

Purely intradural tumours compose 8% of nerve sheath tumours of the first two cervical nerve roots. The percentage of these tumours increases gradually from the high cervical region to the thoracolumbar region. The percentage of strictly extradural tumours and those extending outside the spinal canal gradually decreases from the rostral portion to the caudal portion. These changes in the tumour growth pattern may be explained by the anatomy of the spinal nerve roots, which have a longer intradural component at the more caudal portion of the spinal axis. Thus, the knowledge of the variable anatomical relationship may help, not only in creating surgical strategy for total resection but also to predict radicular dysfunction and weakness [7, 8].

These tumours are usually encapsulated and thus can be excised en-mass. Tumour excision is usually possible with preservation of the normal neural tissue, as noted in this case. Recurrence and malignant transformation are uncommon [9, 10].

These benign tumours have good prognosis and surgical treatment gives relief of the symptoms. Possibility of postoperative neurological deficit due to surgical trauma incurred during dissection, although often transient, should be well explained to the patient before surgery. Although these tumours are a relatively rare cause of nerve root pain, they should be considered in the differential diagnosis of sciatica. This is especially true in patients who fail to respond to an adequate regimen of conservative therapy.
